# Development and Assessment of a Movement Disorder Simulator Based on Inertial Data

**DOI:** 10.3390/s22176341

**Published:** 2022-08-23

**Authors:** Chiara Carissimo, Gianni Cerro, Luigi Ferrigno, Giacomo Golluccio, Alessandro Marino

**Affiliations:** 1 Department of Electrical and Information Engineering, University of Cassino and Southern Lazio, 03043 Cassino, Italy; 2 Department of Medicine and Health Sciences “Vincenzo Tiberio”, University of Molise, 86100 Campobasso, Italy

**Keywords:** Parkinson’s disease, tremor detection, IMU data, simulation, measurement, machine learning

## Abstract

The detection analysis of neurodegenerative diseases by means of low-cost sensors and suitable classification algorithms is a key part of the widely spreading telemedicine techniques. The choice of suitable sensors and the tuning of analysis algorithms require a large amount of data, which could be derived from a large experimental measurement campaign involving voluntary patients. This process requires a prior approval phase for the processing and the use of sensitive data in order to respect patient privacy and ethical aspects. To obtain clearance from an ethics committee, it is necessary to submit a protocol describing tests and wait for approval, which can take place after a typical period of six months. An alternative consists of structuring, implementing, validating, and adopting a software simulator at most for the initial stage of the research. To this end, the paper proposes the development, validation, and usage of a software simulator able to generate movement disorders-related data, for both healthy and pathological conditions, based on raw inertial measurement data, and give tri-axial acceleration and angular velocity as output. To present a possible operating scenario of the developed software, this work focuses on a specific case study, i.e., the Parkinson’s disease-related tremor, one of the main disorders of the homonym pathology. The full framework is reported, from raw data availability to pathological data generation, along with a common machine learning method implementation to evaluate data suitability to be distinguished and classified. Due to the development of a flexible and easy-to-use simulator, the paper also analyses and discusses the data quality, described with typical measurement features, as a metric to allow accurate classification under a low-performance sensing device. The simulator’s validation results show a correlation coefficient greater than 0.94 for angular velocity and 0.93 regarding acceleration data. Classification performance on Parkinson’s disease tremor was greater than 98% in the best test conditions.

## 1. Introduction

In recent years, telemedicine systems have become increasingly popular and important in a rapidly aging world. According to the World Health Organization (WHO), the joint effect of falling birth rates and rising life expectancy will lead one in six people being over 60 by 2030 [1]. In this context, telemedicine plays a key role, because it represents an innovative technology that makes healthcare easier and available to everyone [2]; indeed, doctors can provide healthcare remotely by means of information and communication technology (ICT) [3] (e.g., using video conferences, and through evaluation of medical imaging). A crucial boost to telemedicine’s spread has been provided by the COVID-19 pandemic: in many countries, people were forced to social distance, and all recurrent monitoring activities, such as diabetic monitoring [4], rehabilitation, the evolution of pharmacological treatments, and checking the progression of a pathology [5], were mostly performed with telemedicine. During the pandemic period, the need for remote monitoring not only concerned medical applications but also the sports field: E.g., Cortis et al. [6] performed data collection during a home exercises campaign. The aim was to collect and analyze physiological data such as heart rate, energy expenditure, and oxygen consumption while performing sports activities. For these reasons, the main goals of telemedicine include the possibility of overcoming geographical barriers and connecting users who are distant [7], thereby reducing queues at health centers and shortening waiting lists, and improving the quality of care. A sector where telemedicine is widely applied is represented by the neurodegenerative diseases field. In particular, telemedicine systems could help doctors to monitor patients in daily life and make accurate and early diagnoses. Such objective evaluations will beenhanced by the adoption of specific sensors for data acquisition and computer science efforts for the development of suitable data analysis. To optimize algorithmic development, a large amount of data is generally needed: they could be obtained from a large campaign of experimental measurements involving volunteer patients, although this is not always easy to implement. A second opportunity is represented by the usage of publicly available datasets. In this case, the process of data generation has already been performed. We move towards a third possibility: the creation of a customized data generator, by means of a simulation process. The simulator integrates the whole process, from the raw data coming from sensors to the suitable addition of pathological features. The ability to choose the sensor’s capabilities, metrological features, and noise contribution, and the resolution of the sensing system and kind of motor pathology for the simulation makes the developed simulator a widely applicable tool, since it allows characterizing the algorithms with an arbitrary level of measurement data quality and to generate data for a customizable period of time, avoiding limitations intrinsically present in already available datasets. Specifically, stemming from the authors’ experience in PD monitoring [8,9], this paper presents the development and validation of a pathological movement simulator capable of generating motion and tremor data fully compliant with real measurements performed by IMU devices having specified metrological features.

The main contributions of this work are: (i) the development of a widely-applicable simulator having a two-fold purpose, consisting of replicating the metrological features of several IMU devices and adding signal features to acceleration and angular velocity data to create pathological conditions in terms of movement disorders; (ii) a classification performance assessment which considers measuring device features as variable parameters instead of classical machine learning hyper-parameter optimization; accordingly, the simulator allows engineers to choose their favorite trade-off combination between device cost (and related measurement performance) and classification accuracy; (iii) the building of arbitrarily large datasets, eventually composed of hybrid data, i.e., mixing different measuring devices and obtaining a balanced and cost-effective set-up to propose for physical deployment.

Accordingly, this paper is organized as follows. Section 2 discusses related works of telemonitoring and the main symptoms of PD, adopted as a specific example in this paper. Section 3 describes the adopted methods, i.e., the proposed simulator architecture, implementation, and validation with real data derived from the adopted IMU devices. The validated simulator was used to create pathological and healthy condition data, over which a common machine learning algorithm was tested. Classification results are reported in Section 4. A discussion about classification stability under variable testing conditions, having different data quality, is presented in Section 5. Conclusions and future directions are given in Section 6.

## 2. State of the Art: Remote Monitoring in Neurodegenerative Diseases

The joint effort of the research community concerns the development of methodologies to assess in automatic mode the presence of pathologies, especially in the field of NDD, in the earliest possible way; at the same time, to increase patient’s comfort and involve him/her in the constant monitoring evaluation, researchers are moving towards a completely remote approach. This section provides the reader with the recent advances in the field, reinforcing the motivation for developing a movement disorder simulator to help the community in the optimization of detection and classification algorithms, along with the adoption of suitable wearable devices, whose capabilities should not only be evaluated in terms of communication but also regarding the metrological performance of the adopted instrumentation.

The most challenging NDDs are Alzheimer’s disease (AD), Parkinson’s disease (PD), and Amyotrophic Lateral Sclerosis (ALS) [10]. NDDs are characterized by a continuous decline in cognitive and/or motor functions; even traveling to medical centers for routine follow-ups can become complicated and stressful for patients and caregivers. In this scenario, remote medical approaches are essential to ensure monitoring and medical care: such methods have already been widely used [11]. To get remote data to allow medical staff to perform continuous monitoring of patients, objective methods and suitable instrumentation are needed. Monitoring devices should be accurate, minimally invasive, and easy to wear or adopt by the patient in autonomous or semi-autonomous modes. As concerns the measuring devices for motor symptoms, there are different sensors and techniques that can be used to capture human movements. Firstly, optical measurements [12] are widely used for the advancements in image processing and their easiness of use. As an example, the leap motion system is used in [13] to detect upper limb motor skills in Parkinson’s disease. The depth camera system is economic and easy to use in movement detection applications, but it presents occlusion issues during upper limbs’ joint movement or their sudden exit from the field of view [12]. Another study proposed a magnetic, low-cost, and scalable system to monitor the evolution of PD [8]. In particular, the system focused on two parameters: tremor and hands trajectory, and an anchored magnetic measuring system was used to assess the position of the hand in a 3D limited space and a single accelerometer, with a sampling frequency of 100 Hz to detect tremor. In order to simplify the measurement system and converge towards simpler hardware solutions, the usage of wearable sensors could be a viable solution. The smaller and simpler the adopted sensors, the heavier and more complex the processing algorithms to get movement recognition. Furthermore, the deterministic relation between obtained measurements and the patient’s state of health could be difficult to infer in a straightforward approach. Therefore, while remote telemedicine is already widely spreading, the research for discriminant, sensitive, specific algorithms to get health status from raw data is always progressing. In this direction, wearable sensors and artificial intelligence can represent an interesting solution for the objective and quantitative evaluation of body movement. These issues are widely addressed in the literature, and so there are different measurement systems used to capture and classify motion. As an example, in [14], the authors presented the results of a pilot study to evaluate the possibility of using accelerometer data, acquired with a sampling rate of 100 Hz, and a camera to video record movement activity, to find and study motor complications in patients with PD. They used a support vector machine (SVM) classifier to estimate the severity of tremor, bradykinesia, and dyskinesia from accelerometer data. Another application is described in [15], where the authors wanted to distinguish PD tremor from essential tremor. They used six inertial measurement units (IMUs) placed on several patient’s body parts, and for the classification, they implemented different machine learning algorithms, i.e., neural networks, SVM, k-nearest neighbor, decision tree, random forest, and gradient boosting; the best performance was obtained with the SVM technique, achieving 89% accuracy. Papadopoulos et al. [16] focused their work on the problem of automatically detecting PD tremors from IMU data collected in the wild via a smartphone. They introduced a new dataset of accelerometer recordings from both PD patients and healthy subjects, captured outside laboratory conditions. To acquire tremor annotations for each user, they used the widely recognized Unified Parkinson’s Disease Rating Scale (UPDRS) [17]. Their method operates on accelerometer signals only during phone call events, and the authors used a multiple-instance learning approach for movement disorders classification. The sampling rate used to acquire the data depends on the mobile phone used by the individual subjects. In the aforementioned works, the authors used different machine learning algorithms to classify PD movement disorders: to perform accurate tests, it is always necessary to collect enough data in different operating conditions. Therefore, the involved patients have to repeat the required tasks more than once on different days, at home or in a clinical environment [18], so it takes time before a validated dataset is created. Accordingly, a large amount of data from different patients is needed, and due to voluntary availability issues and a normative framework that takes long periods to get authorizations, such amounts of measurement data are rarely available. To this end, the authors focused their efforts on developing a movement disorder simulator that allows one, whenever properly set with the required information, to output validated data for different movement disorders and to take into account sensing devices’ measurement capabilities. In this case, a software framework fed by a limited amount of real data could lead to arbitrarily long and specific data generation, able to describe several pathological and healthy conditions, customizable in terms of measurement acquisition accuracy, and easily portable and reproducible with different levels of disease. The developed simulator works on inertial data coming from one or more IMU devices, derived from specific trajectories which could be retrieved from standardized tasks that doctors require their patients to perform. The simulator’s added value is to corrupt such data by means of suitable perturbations that are typical of the considered movement disorder. The choice to use IMU data as the basis for the simulation is due to the large amount of scientific research adopting inertial devices for healthcare purposes [19,20]. Although general, to give practical proof of its working principle, the developed simulator is here presented with a focus one of its specific features: the production of PD tremor-related data. In detail, main motor symptoms associated with PD are represented by tremor, bradykinesia, muscle rigidity, and postural instability [21], which are normally diagnosed during classic clinical examinations through home diaries, neurological information, and standardized tests. These classical methods are influenced by subjective aspects, medical experience, and the patient’s ability to notice the presence of a specific symptom [22]. The limitations described can be overcome through the use of objective evaluation techniques, the development and optimization of which are possible with a large dataset that can be realized using a well-designed simulator.

One of the main motor disorders of PD is tremor, which decreases the quality of life by interfering with daily activities [23]. Tremor is defined as a rhythmic involuntary and oscillatory movement that occurs in body parts such as hands, legs, vocal folds, the trunk, or head, and it is a clearly visible motor phenomenon in most cases [24]. Tremors are classified by the Consensus Statement on the Classification of Tremor [25], according to the behavior exhibited during their appearance. In particular, we can distinguish resting, postural, or action tremors. Rest tremor (RT) happens when a body part is relaxed; conversely, postural tremor (PT) is achieved when a body part is held straight out from the body in a stable position against gravity; finally, kinetic tremor (KT) occurs when an action is performed by voluntarily contracting a muscle [26]. The RT is classified as a tremor with a frequency between 4 and 7 Hz [27] and occurs in 70% of patients with PD, and it tends to disappear with voluntary movements [27]. KT causes higher disability in patients then other types of tremors; it occurs at a frequency of around 9 Hz, which is higher than in the case of RT. Finally, PT occurs at a frequency of between 5 and 8 Hz [8]. Today, neurologists assess the severity of these disorders by administering psychometrics and cognitive tests, and then evaluating them using the UPDRS standardized scale [28]. It is the standard scale most commonly used by specialists during current clinical examinations to quantify the severity of various elements in PD in motor and non-motor issues. The clinical evaluations are based on experience and observation by specialists. It is important to find and test an objective system that can analyze movement disorders and help neurologists to do correct diagnoses, especially in the first phase of the disease when there are mild symptoms. In detail, to detect tremors and make treatment recommendations, doctors use a short observation time in the clinical setting and diaries filled by patients. These procedures can yield a coarse rating of the tremor due to a lack of objective measures of movement, and an incorrect diagnosis can lead to unsuccessful therapy [29]. Several research groups, as described in this Section, have proposed objective methods to detect and quantify tremors using wearable sensors, which are non-intrusive systems enabling one to perform quantitative, objective, and continuous measurements of movement. Among these, IMUs play an important role: thanks to their small size and easy handling, patients can use the devices directly at home during normal daily activities. For these main aspects, in this work, inertial sensors are considered to do objective measurements and detect PD tremors.

## 3. Methods: The Proposed Simulator

In the NDD field, particularly in motor detection, to analyze and classify specific pathologies it is necessary to involve patients and collect a large number of data in order to generalize the study and obtain reliable performance. In this scenario, in order to quickly get the required large amount of data and assess the measurement procedure and classification phases, it is valuable to have a software tool, namely, a simulator, able to generate specific pathological movements. The simulator must be able to generate and reproduce signals and movements similar to real ones: to this end, a preliminary validation phase is necessary. Its implementation may require the definition of a complex mathematical model or be based on measurements from the field, stemming from purpose-specific acquired values or long-term monitoring studies. In both cases, a comparison between the generated data and measurements from the field is always needed to validate the process. The simulator’s description and the related validation phase are reported in the following subsections.

### 3.1. The Proposed Architecture

**Figure 1:**
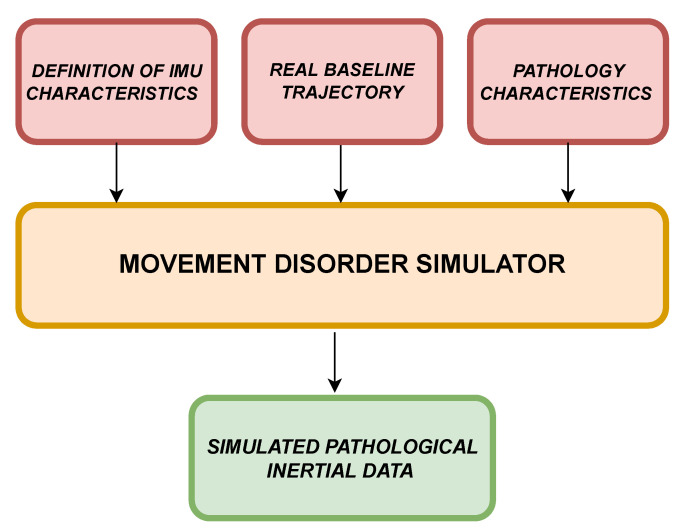
Simulator block diagram.

Figure 1 shows the block diagram of the movement disorder simulator. Three inputs are required to generate pathological movements: IMU characteristics, real inertial data related to a specific trajectory, and pathology characteristics. In the first case, once the real device to be simulated has been identified (in this case, a nine-axis IMU), it is necessary to define the metrological characteristics of the simulated sensor. For this, it is necessary to set parameters related to (i) hardware characteristics, (ii) noise, and (iii) environmental factors [30].

With regard to the hardware characteristics, several parameters can be defined:

- the vertical resolution of the analog-to-digital converter ($r_{ADC}$), which influences the digitization process of the acquired data;
- the axis misalignment value for the three-axis IMU considered ($a_{MIS}$);
- the constant bias value ($b_{VAL}$), which influences all measurements by altering their average values and which is generally attributable to hardware defects.

As far as (ii) is concerned, the sensor measurements could also be affected by several random noises ($rnd_{NOISE}$):

- white noise;
- random walk, i.e., the amount of Brownian noise;
- bias instability, which concerns the level of pink or flicker noise in the measurement.

The simulator makes it possible to define which subset of them should be used to take into account the metrological performance of the considered sensor.

Finally, with regard to the effects of the environment (third category), the following quantities can be set:

- bias temperature ($b_{TEMP}$), defined as the difference from the predefined operating temperature;
- temperature scaling factor ($sf_{TEMP}$), which considers the error due to variations in the operating temperature.

(1) $$IMU_{MODEL}=f\left(r_{ADC},a_{MIS},b_{VAL},rnd_{NOISE},b_{TEMP},sf_{TEMP}\right);$$

In Equation (1), the function *f*, taking as input the aforementioned parameters, provides a model ($IMU_{MODEL}$), needed to perturb the nominal trajectory.

At this stage, the real baseline trajectory has to be considered. Accordingly, two possible solutions are theoretically available: acquire such data from the field or generate them digitally. In the case of trajectory generation, Equation (2) has to be considered. It deals with imposed coordinates and orientation (subscript "q" stands for "quaternion" values) and the IMU model found. The second solution is more general, since it allows generating data considering whatever IMU model where the information required by Equation (1) is available. As regards the first solution, which was adopted for the experimental validation, it is strictly important that the acquisition from the field is performed with a reference IMU, i.e., a device that has far better metrological performance than the ones it is needed to simulate. In this way, the acquired trajectory can be assumed as ideal.

(2) $$T\left(t\right)=g\left(\vec{x},\vec{y},\vec{z},\vec{x_{q}},\vec{y_{q}},\vec{z_{q}},\vec{w_{q}},IMU_{MODEL}\right);$$

Finally, having identified the pathology to be simulated, it is necessary to provide a mathematical description of the main objective motor effects provoked by the pathology, to be used as baseline trajectory perturbation.

(3) p(t)=h(fr,a,T,n);

Applying Equation (3), it is possible to get the perturbed and pathological trajectory. The parameters of the *h*-function are: frequencies (fr), amplitudes (*a*), perturbed trajectory (*T*), and eventual adding noise sources (*n*) that could be defined by the pathology itself.

The simulator output, outputting IMU typical quantities, is characterized by simulated pathological inertial data, expressed as a 9-axis matrix, containing acceleration, angular velocity, and magnetic field as time-domain profiles.

To implement the *h*-function, it was necessary to consult and study the literature on movement disorders in Parkinson’s disease. For example, in works [31,32], the characteristics of tremor in terms of typical frequency ranges, typical energy in the frequency domain, and accelerometer waveforms during a tremor event were consolidated.

### 3.2. Experimental Validation

In this application, the proposed simulator was specifically employed to reproduce inertial data characterizing PD, particularly tremors occurring in the upper limbs during a linear trajectory in a 3D space. A validation procedure was carried out to evaluate its suitability to faithfully generate IMU data in nominal conditions, i.e., without the disease effects by comparing generated data with real ones.

**Figure 2:**
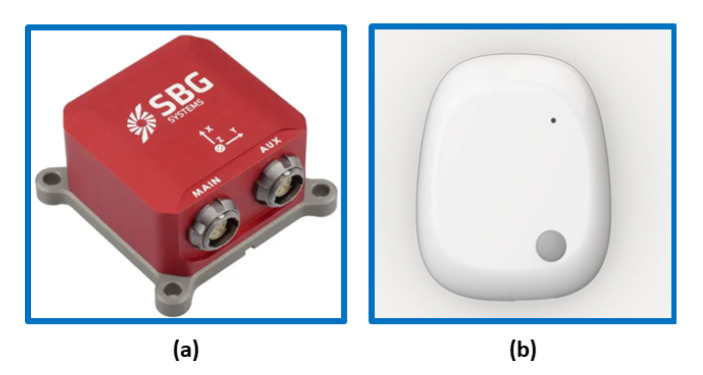
IMU devices used for validation: (**a**) SBG Ellipse-E; (**b**) MetaMotionR.

Two different sensors were used for the evaluation:

- SBG Ellipse-E (SBG) made by SBG Systems company (Figure 2a) [33];
- MetaMotionR (MMR) made by mbientlab company (Figure 2b) [34].

Regarding the SBG Ellipse-E [33], it is a compact device with a high-performance inertial navigation system (INS). It includes a MEMS-based IMU and runs an enhanced extended Kalman filter (EKF) that fuses inertial and aiding information in order to obtain accurate real-time orientation and navigation data. It is capable of supplying data with a maximum frequency of 1000 Hz [33]. The other considered sensor is the MetaMotionR (MMR) [34], which is a wearable device capable of offering real-time and continuous monitoring of motion and environmental sensor data. In this case, the maximum frequency of data output is 100 Hz. It is widely used for scientific studies on patients in clinical settings. Each sensor has an onboard tri-axis accelerometer, a gyroscope, and a magnetometer with a 16-bit A/D converter. Table 1 shows the measurement parameters used to set the devices.

#### 3.2.1. The Simulator Validation

**Figure 3:**
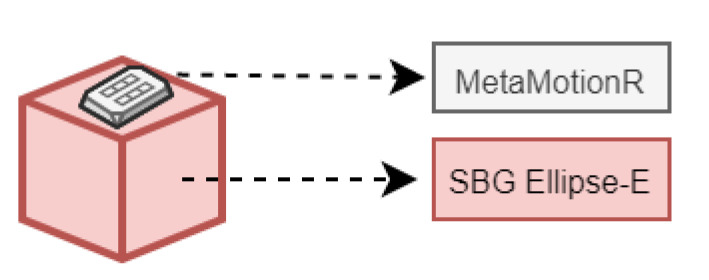
SBG and MMR measurement configuration.

The aim of the current subsection was to carry out static and dynamic tests with real IMU sensors to validate the simulator’s capability to generate IMU-like data and to assess its basic performance. The idea was to compare the real data acquired during movement tests with simulator inertial results in order to evaluate their measurement compatibility, i.e., to state if data differences are only due to random factors characterizing the sensors’ measurement uncertainties or if specific biases or deterministic phenomena intervened during the measurement process. The usage of two IMU sensors was due to the need to have a reference instrument (namely, SBG) that feeds the simulator (providing inertial data from real baseline trajectory) and a second IMU sensor (MMR), whose data, acquired during such a trajectory, should be compatible with those obtained from the simulator, programmed to generate MMR-like data (Figure 3).

**Figure 4:**
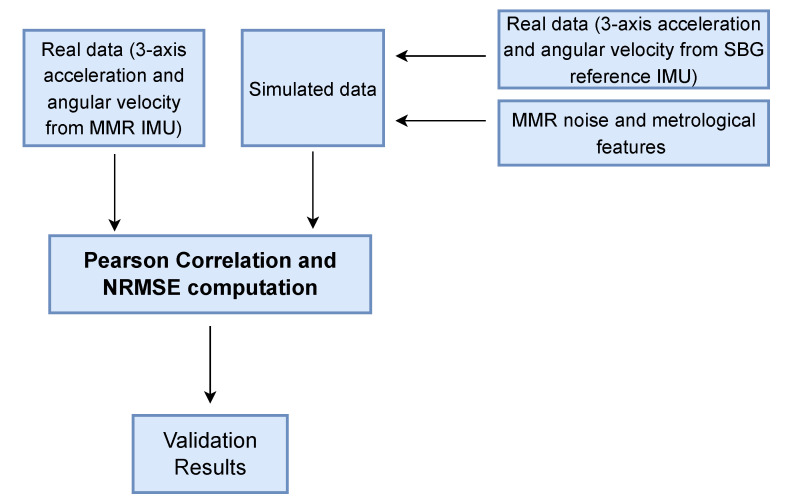
Block diagram to perform the validation procedure.

**Figure 5:**
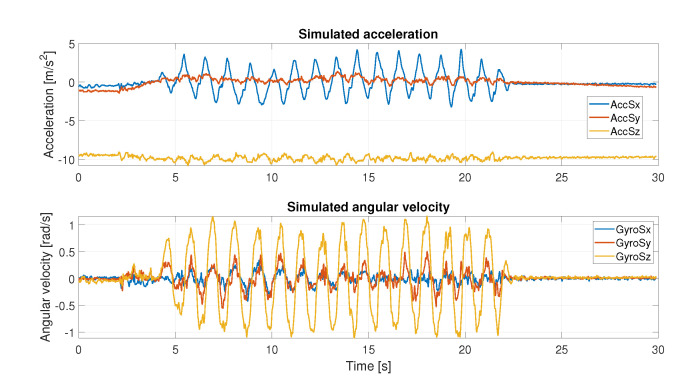
Simulated acceleration and angular velocity recording during horizontal movement.

Subsequently, dynamic tests were performed: the sensor couple was placed on the back of an operator’s hand (to assume a position that is typical of a smart watch’s placement), and several movements in the 3D space were performed. During the test phase, inertial data from SBG and MMR were collected and subsequently used in the validation process, as shown in Figure 4. The simulator received as input the inertial data from SBG. It perturbs the input inertial data using MMR characteristics (Table 1) to obtain simulated IMU values as output. As an example, Figure 5 displays the simulated accelerometer and angular velocity data during one of the horizontal movements.

#### 3.2.2. Results Validation

To verify the reliability of the simulation results, two suitable figures of merit are identified: Pearson’s correlation coefficient and root mean square error (RMSE), described by Equations (4) and (5), respectively. They were adopted to evaluate the similarity degree between simulated and real acquired data.

(4) $$\rho_{xy,\%}=\frac{E[\left(X-\mu_{x}\right)\left(Y-\mu_{y}\right)]}{\left(\sigma_{x}\sigma_{y}\right)}\times 100;$$

(5) $$RMSE_{\%}=\sqrt{\frac{1}{n}\Sigma_{i=1}^{n}{\left(X_{i}-Y_{i}\right)}^{2}}\times 100;$$

In Equations (4) and (5), *X* corresponds to MMR signal samples, and *Y* represents the simulated inertial data. As for Equation (4), $\sigma_{x}$ and $\sigma_{y}$ are the standard deviations of X and Y; the mean values of X and Y are represented by $\mu_{x}$ and $\mu_{y}$, respectively; *E* is the expectation operator.

Before computing these parameters, it was necessary to normalize data. The min-max normalized method [35] was used.

**Figure 6:**
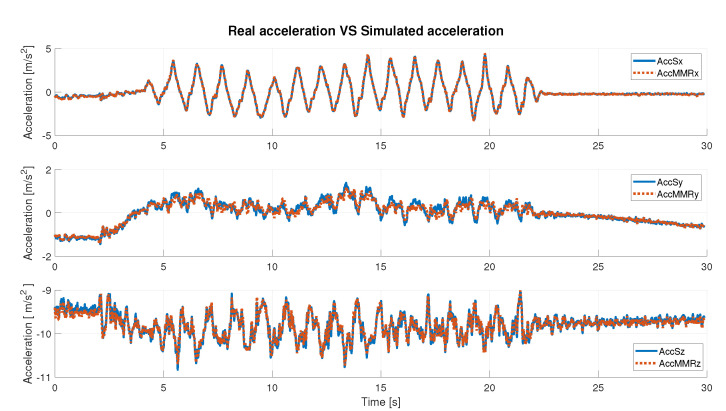
Real acceleration data vs. simulated acceleration data.

**Figure 7:**
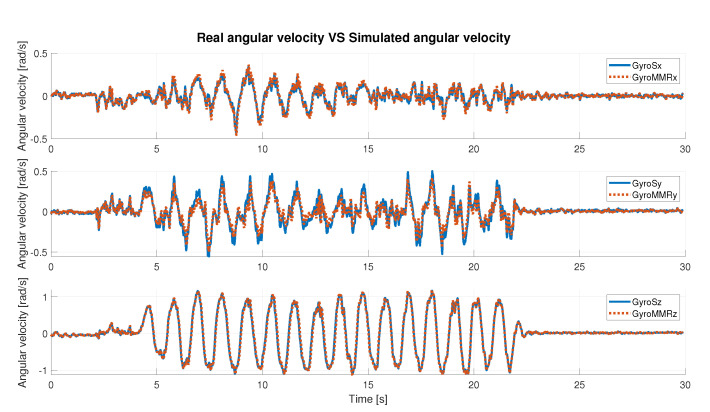
Real angular velocity data vs. simulated angular velocity data.

In Table 2 and Table 3, the obtained results are described, considering angular velocity and acceleration data, respectively. The results are distinguished axis by axis and consider the absolute value (**abs**) of the signal. Qualitatively, the results can also be seen in the Figure 6 and Figure 7, where simulated IMU data are superimposed on the real data. Under defined operating conditions, the simulator is able to reproduce inertial data with a high level of reliability.

Under any condition, the Pearson’s coefficient is greater than 94% in the angular velocity comparison; instead, it is greater than 97% in the acceleration comparison, axis by axis. It slightly decreases when the **abs** is considered. In detail, it is possible to observe that for the acceleration data (Table 3), the best value is along the x-axis (99.88%), whereas in the case of the gyroscope (angular velocity, Table 2), it is along the z-axis (99.35%). Considering the correlation results in terms of the absolute value of the signal, the highest value was obtained in the case of the simulated angular velocity, and it is greater than 98%.

The RMSE is generally bounded from above by 6% (5.66% for acceleration along y-axis). As the best-observed values for angular velocity data the lowest RMSE is 2.77%, achieved on the z-axis, and for acceleration data, the lowest error is along the x-axis and is equal to 0.99%. Finally, the error computed on the absolute value, in either case, is slightly greater than 4%. These results show a high compatibility level between simulated and real data.

### 3.3. Pathological Movement Generation

Among Parkinson’s motor disorders, tremor is diagnosed in more than 70% of patients. To the best of our knowledge, there is no objective method to distinguish different types of tremors, but neurologists use patient history and physical examinations evaluated through UPDRS metrics [36]. PD tremor is the most recognized sign, and although it could be not life-threatening, it surely influences normal daily activities and reduces patients’ quality of life [37]. Objective measurements and classification systems, such as the joint use of IMU and machine learning algorithms, can be valuable tools to help clinicians make increasingly accurate diagnoses. In this section, data associated with PD tremors, generated by means of the proposed simulator, are reported and discussed.

#### Adopted Tremor Typologies

Among the many possible typologies of tremor, this article focuses on the most common three types of hand tremor: resting tremor (RT), postural tremor (PT), and kinetic tremor (KT); Table 4 summarizes their main features [38], as already mentioned in Section 2.

To replicate the pathological typical assessment movements, three reference tests were identified from the UPDRS document [17].

- Test for the identification of postural tremor of the hands: the subject’s arms are stretched out in front of the body with the palms down, the wrist should be straight and the fingers should not touch. (Section 3.15);
- Test for the identification of kinetic tremor of the hands: this test uses the finger-to-nose technique. Specifically, the subject starts with the arm outstretched and must then perform at least three finger-nose movements with each hand extending as far as possible until it touches the examiner’s finger. (Section 3.16);
- Test for identification of resting tremor: the subject sits quietly in a chair with hands resting on the arms of the chair and feet resting on the floor. This position should be held for 10 s without any other directions. (Section 3.17).

These tasks are administered by the neurologist in classical examinations. The replication of such tasks for our purposes has been carried out by performing the following phases.

- Before generating the pathological tests, baseline tests for each task were carried out. The operator placed the SBG sensor on the top of the hand and performed the tests as described in the guide. This first collection of data was considered free of any pathology.
- Subsequently, tremors to these traces were added: they were modeled analytically as multisine signals whose frequency and amplitude range [17] were derived from [32] and added to the baseline perturbed traces (the MMR-like signals are always generated using the simulator). On each baseline inertial trace, several 2 s pathological tremors at disjoint intervals were superimposed for each axis. For each test, 1000 trials of 60 s duration were generated, each containing five 2 s tremor time segments. Tremor was generated for each interval randomly in terms of frequency and amplitudes, although within the recommended values, in order to generate a widely general dataset.

**Figure 8:**
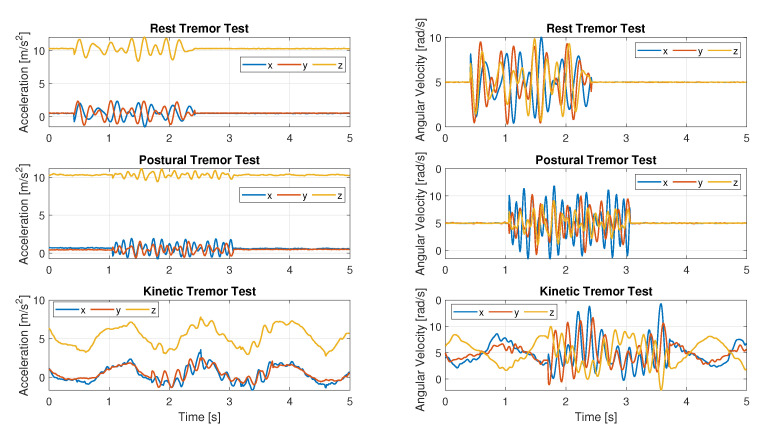
Simulated pathological data: acceleration data on the left; angular velocity data for the same tests on the right.

Figure 8 displays an example of simulated accelerometer and gyroscope data for all considered tasks.

## 4. Results: Tremor Classification

In this section, a tremor classification approach is proposed, and it is based on pathological data generated by the aforementioned validated simulator. In detail, the adopted machine learning tool is described at first, and the obtained classification results are reported next.

### 4.1. The Machine Learning Tool

The classification phase is run by adopting an ML algorithm fed with the generated dataset. Among all available ML techniques in the Matlab^TM^ environment, the Fine Tree (FT) tool was chosen as a result of preliminary tests where training accuracy was adopted as a figure of merit. FT must be able to distinguish four classes: no tremor (Class 1), rest tremor (Class 2), postural tremor (Class 3), and kinetic tremor (Class 4). To recognize tremor and voluntary movement, 11 features were selected. Such choice was derived from the literature related to classification in human activity and motor symptoms’ framework [32,39,40]. 

Among these, eight features belong to time domain processing:

- Mean [39];
- Averange [39];
- Square sum of data under 25th percentile;
- Squared sum of data under 75th percentile;
- Low pass energy (below 2 Hz signal energy, to identify voluntary movement) [40];
- High pass energy (over 2.5 Hz signal energy, to identify involuntary movement) [40];
- Lag of first autocorrelation peak (to find the dominant frequency of the involuntary movement) [32];
- Height of first peak in autocorrelation (to discriminate periodic movements from aperiodic ones) [32];

and 3 features are related to the frequency domain [41]:

- Maximum frequency in the spectrum;
- Sum of amplitude values of frequency components below 5 Hz;
- Number of peaks in the same frequency spectrum interval;

All features are calculated over a 2 s window, with 1 s overlap between consecutive windows [32]. For good performance of the ML algorithm, it is necessary to normalize the features’ input data: before moving on to classification, dataset normalization was carried out by means of a Z-score feature scaling method [42].

The classification stage involved two main analyses. The first analysis adopts one only dataset, where 70% of the data were used to train the tool, and the remainder were used for the test phase. Part of the first step was also to look for the effects of reducing/increasing the measurement axes for the accelerometer and/or gyroscope, i.e., to consider smaller amounts of data, on the classification performance.

A second analysis concerning a different goal: to test the algorithm on data derived from different sensors with respect to those used in the training phase and to calculate the obtained performance in order to establish possible relations between metrological features of sensors acquiring training data (that can be performed once) and those related to instrumentation gathering test data (eventually, in real-time). This second test is particularly relevant, since we wanted to understand if low-cost IMU sensors could be adopted for real-time monitoring, regardless of the sensors adopted to train the classification algorithm. To take account of the performance obtained during the test phase, accuracy, precision, recall, and F1-score were calculated.

### 4.2. Classification Metrics and Performance

To analyze the obtained classification results, we needed to define three specific figures of merit, namely, precision, recall, and F_1_-score. They are described in Equations (6)–(8), respectively.

(6) $$Precision\left(p\right)=\frac{TP}{TP+FP};$$

(7) $$Recall\left(r\right)=\frac{TP}{TP+FN};$$

(8) $$F_{1}-Score\left(F_{1}\right)=2\frac{p\times r}{p+r};$$

(9) $$Accuracy=\frac{TP+TN}{TP+TN+FP+FN};$$

where TP, TN, FP, and FN represent true positive, true negative, false positive, and false negative cases by their common definitions, respectively.

**Figure 9:**
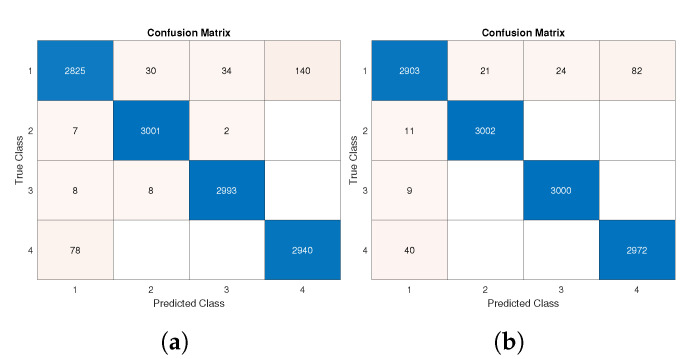
Confusion matrix of test phase: (**a**) considering acceleration data from all axes; (**b**) considering acceleration and angular velocity data from all axes.

The total number of samples was around 10,000. As stated before, 70% of them were used for training and the remaining ones for testing. We present results in two distinct ways: confusion matrices and aggregate values of the adopted figures of merit. In detail, Figure 9 reports the obtained confusion matrices for two distinct cases regarding the data for training and testing: accelerometer values only (Figure 9a) and the joint use of accelerometer and gyroscope data (Figure 9b).

As is widely known, the better the performance, the greater the value on the confusion matrix’s main diagonal. A very high TP positive rate was obtained for all classes, and particularly for Classes 2 and 3.

Such results are confirmed by Table 5 and Table 6, where the highest value for each figure of merit is reported in bold. A fast comparison between the cited tables proves how the joint adoption of accelerometer and gyroscope data slightly increases performance values.

In detail, the best improvement was obtained in the *recall* parameter in the case of Class 1, where 93.27% was obtained with accelerometer data only (Table 5), whereas 95.81% is achieved with the additive support of gyroscope data. Taking the test accuracy as an aggregate performance index, a 0.99% (from 97.46% to 98.45%) improvement was achieved by using more data sources.

A specific mention is deserved by Table 7. In order to exploit as few data as possible and remain in the first classification step, we analyzed the effect of reducing the amount of data by limiting the number of acquisition axes both for the accelerometer and for the gyroscope instruments. Surprisingly, although general performance worsening is visible, reducing the data amount to one or two axes did not heavily impact the very high scores achieved by the defined performance indexes. Performance levels decreased a few percentage points, thereby still ensuring good classification accuracy for the required types of tremors. Still, training and testing were performed on the same dataset, although subdivided in the aforementioned way.

## 5. Discussion: Classification Performance Stability under Data Quality Variation

**Figure 10:**
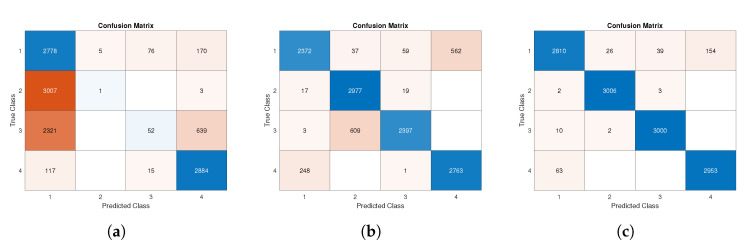
Confusion matrix of test phase: (**a**) test data according to best/worst specifications; (**b**) test data according to worst/best specifications; (**c**) test data according to worst/worst specifications.

Another key factor we wanted to explore is the possibility of having datasets generated from different sensors for training and testing phases. In particular, it is desirable to understand how the parameters of the sensors could affect the classification results whenever an uneven set of data is fed to the classifier during the learning phase and the following test. The results presented in this section check if the most common situation is viable for the application purposes—i.e., the best accurate sensors are available to train and tune the classifier—but less accurate devices are generally available during normal monitoring activities due to cost constraints and the large variety of IMU sensors available on the market. In any case, to have a full report of the possible cases, several training/testing combinations were considered. In detail:

a. Training and testing with data coming from the same high-performance sensor (best/best);
b. Training with high-performance sensor data and testing with lower-level sensor data (best/worst);
c. Training with lower-level sensor data and testing with high-performance sensor data (worst/best).
d. Training and testing with data coming from the same lower level sensor data (worst/worst);

In all combination tests, data from the three-axis accelerometer and three-axis gyroscope were considered. Although in Section 4.2 we proved how the reduction of involved axes does not critically reduce the performance indexes, here we analyze a different effect, and therefore, to avoid possible joint causes, we restored the original maximum number of available data, specifically in terms of sensors’ axes. The results and performances in the best condition (case (a)) are described in Section 4.2. The results obtained in the test (c) show the case of low-quality data training and high-quality data testing. Comparing Table 8 with best case (Table 6), it is possible to observe a general performance drop: in particular, for Class 3 of index recall a decrease in 19.81% was recorded (from 99.47% to 79,66%), and the test accuracy went down by 11.34% (from 98.45% to 87.11%). These results are also visible in Figure 10b, where a test confusion matrix is shown. In the case worst/worst (d), it can be seen that by training and testing the network with the same lower-level sensor data, the test phase performances were similar to those obtained in case (a); indeed, the difference between tests’ accuracies was 0.93% (for details, see Table 9 and Figure 10c).

A broader discussion of the case best/worst test results is necessary. Firstly, performance degradation is visible when comparing the confusion matrix in Figure 9 with the one shown in Figure 10a. The worst results were recorded for the identification of Classes 2 and 3 especially. Both classes, in most cases, were wrongly predicted as Class 1.

The aforementioned results were confirmed by indexes reported in Table 10, where a general decline in performance can be seen. Comparing Table 10 with Table 6, we can see that the worst decrease was obtained in the $F_{1}$-score index, especially for Class 2, where a radical reduction was recorded. The test accuracy also went from 98.45% (Table 6) to 47.36% (Table 10). The highest number of misclassifications was recorded for Classes 2 and 3, with all indices showing values below 17% for class 2 and below 37% for Class 3.

According to the reported data, we may say that the usage of uneven datasets for training and testing phases is generally not advised, since the performance degradation is quite critical. Anyway, there are specific worsening cases, such as only the reduction of the quantization bits (case Table 8), where the adoption of different sensors still warrants good performance indexes. In our investigation, the number of quantization bits had a more negligible effect on the performance than the worsening of all noise levels.

## 6. Conclusions and Future Directions

The paper presented the development, validation, customization, and adoption of a movement disorder simulator, capable of generating reliable acceleration and angular velocity data related to healthy and pathological states for patients possibly affected by Parkinson’s disease. In particular, taking into account commercial IMU sensors, the aim of the tool consists of generating real-like data to create arbitrarily large datasets to be exploited by researchers in the field of NDD automatic diagnosis to test and tune their classification algorithms. To evaluate the reliability of generated data, a validation set-up was developed, and real acquired and simulated data were compared by means of two suitable figures of merit, which have proved a high agreement level. Subsequently, a classification stage was developed by using the fine tree algorithm on data that were suitably generated, and results were evaluated through classical performance indexes in the field of machine learning. Performance scores generally equal to or greater than 90% were achieved in most cases, whenever homogeneous (different but acquired with the same sensors) data were adopted for training and testing. Two further analyses were presented in the paper: the effects of the number of axes and sensors (accelerometer, gyroscope) and the adoption of uneven data for training and testing to the classification results. The obtained results showed slight performance worsening when the number of adopted axes decreased and interesting relations between metrological features of the sensors and classification results. The paper is intended to give useful information for the choice of proper IMU sensors (metrological aspects) to adopt for NDD monitoring, to prove the suitability of such sensors for acquiring useful data for automatic diagnosis of Parkinson’s disease based on tremor classification; and for the adopted classification algorithm, the work aimed to prove how uneven datasets are generally to be avoided, but in specific cases, acceptable performance can be achieved, whenever only specific features change (e.g., quantization bits). The next validation step of the proposed simulator was to compare its generation with healthy and pathological movements coming from real patients, in order to understand how IMU data should be adapted to different patients’ statuses, such as age, disease gravity, and eventually comorbidities. Another envisioned future direction is to move the simulator to an open-source programming platform and adopt free programming languages to make not only the datasets but the whole simulator architecture freely available to all researchers working in this field.

## Figures and Tables

**Table 1:** Sensors’ main parameters.

	SBG Ellipse-E	MetaMotionR
**Sample Rate**	1000 Hz	100 Hz
**Resolution**	16 bit	16 bit
**Accelerometer Range**	±16 g	±16 g
**Gyroscope Range**	±1000°/s	±2000°/s
**Accelerometer** **Noise Density**	57 $\mu g/\sqrt{\mathrm{Hz}}$	180 μg/$\sqrt{\mathrm{Hz}}$
**Gyroscope** **Noise Density**	0.0025°/s/$\sqrt{\mathrm{Hz}}$	0.0070°/s/$\sqrt{\mathrm{Hz}}$

**Table 2:** Pearson’s correlation and RMSE computed for real and simulated angular velocity data.

	Pearson’s Correlation [%]	RMSE [%]
**x**	95.70	3.51
**y**	94.30	4.98
**z**	99.35	2.77
**abs**	98.38	4.40

**Table 3:** Pearson’s correlation and RMSE computed for real and simulated acceleration data.

	Pearson’s Correlation [%]	RMSE [%]
**x**	99.88	0.99
**y**	97.21	5.66
**z**	97.94	4.59
**abs**	93.64	4.43

**Table 4:** Adopted types of tremors: main features.

Types of Tremor	Clinical Features
**Rest Tremor**	RT occurs when there are not voluntary movements and the limbs are at rest and supported against gravity.
**Postural Tremor**	PT is define as an action tremor, it is occurs when a position is maintained against gravity.
**Kinetic Tremor**	KT is an action tremor, which appears during voluntary movement

**Table 5:** Classification performance results computed on acceleration data only (considering all axis).

Class\Metrics	*p*	*r*	$F_{1}$
No Tremor (1)	96.81%	93.27%	95.01%
Postural Tremor (2)	98.75%	**99.70%**	**99.22**%
Rest Tremor (3)	**98.81%**	99.47%	99.14%
Kinetic Tremor (4)	95.45%	97.42%	96.43%
**Test Accuracy**	97.46%		

**Table 6:** Classification performance results computed on acceleration and gyroscope data (considering all axis).

Class\Metrics	*p*	*r*	$F_{1}$
No Tremor (1)	97.98%	95.81%	96.88%
Postural Tremor (2)	**99.31%**	99.63%	**99.47%**
Rest Tremor (3)	99.21%	**99.70%**	99.45%
Kinetic Tremor (4)	97.31%	98.67%	97.99%
**Test Accuracy**	98.45%		

**Table 7:** Classification performance results in different conditions for accelerometer and gyroscope.

	accelerometer x-axis only			accelerometer xz-axes		
**Class\Metrics**	*p*	*r*	$F_{1}$	*p*	*r*	$F_{1}$
No Tremor (1)	96.24%	91.15%	93.62%	97.19%	91.19%	94.09%
Postural Tremor (2)	**97.91%**	98.17%	98.04%	98.52%	**99.70%**	**99.11**%
Rest Tremor (3)	97.49%	**99.27%**	**98.37%**	**98.75%**	99.44%	99.09%
Kinetic Tremor (4)	94.21%	97.25%	95.71%	93.77%	97.81%	95.75%
**Test Accuracy**	96.71%			97.03%		
	**accelerometer and gyroscope: x-axis**			**accelerometer and gyroscope: xz-axes**		
**Class\Metrics**	*p*	*r*	$F_{1}$	*p*	*r*	$F_{1}$
No Tremor (1)	97.57%	92.77%	95.11%	96.54%	94.78%	95.65%
Postural Tremor (2)	**97.92%**	98.41%	**98.16%**	**98.58%**	99.20%	98.89%
Rest Tremor (3)	97.32%	98.61%	97.96%	98.55%	**99.40%**	**98.98**%
Kinetic Tremor (4)	95.95%	**98.94%**	97.42%	97.19%	97.51%	97.35%
**Test Accuracy**	97.19%			97.72%		

**Table 8:** Classification performance results computed on acceleration and gyroscope data: in the training phase 8 bit sensors were used, and for the test phase 16-bit sensors (worst/best).

Class\Metrics	*p*	*r*	$F_{1}$
No Tremor (1)	89.85%	78.28%	83.67%
Postural Tremor (2)	82.17%	**98.81%**	**89.72**%
Rest Tremor (3)	**96.81%**	79.66%	87.40%
Kinetic Tremor (4)	83.10%	91.73%	87.20%
**Test Accuracy**	87.11%		

**Table 9:** Classification performance results computed on acceleration and gyroscope data: in the training phase and test phase, 8 bit sensors with 10% worse noise were used (worst/worst).

Class\Metrics	*p*	*r*	$F_{1}$
No Tremor (1)	97.40%	92.77%	95.03%
Postural Tremor (2)	**99.17%**	**99.83%**	**99.45**%
Rest Tremor (3)	98.62%	99.60%	99.11%
Kinetic Tremor (4)	95.04%	97.91%	96.46%
**Test Accuracy**	97.52%		

**Table 10:** Classification performance results computed on acceleration and gyroscope data: in the training phase, 16 bit sensors were used, and for the test phase, 8 bit sensors with 10% worse noise were simulated (best/worst).

Class\Metrics	*p*	*r*	$F_{1}$
No Tremor (1)	33.78%	91.71%	49.38%
Postural Tremor (2)	16.67%	0.03%	0.07%
Rest Tremor (3)	36.36%	1.73%	3.30%
Kinetic Tremor (4)	**78.03%**	**95.62%**	**85.94**%
**Test Accuracy**	47.36%		

## Data Availability

Not applicable.
